# Informing the development of Australia’s National Eating Disorders Research and Translation Strategy: a rapid review methodology

**DOI:** 10.1186/s40337-022-00556-3

**Published:** 2022-03-04

**Authors:** Phillip Aouad, Emma Bryant, Danielle Maloney, Peta Marks, Anvi Le, Haley Russell, National Eating Disorder Research Consortium, Phillip Aouad, Sarah Barakat, Robert Boakes, Leah Brennan, Emma Bryant, Susan Byrne, Belinda Caldwell, Shannon Calvert, Bronny Carroll, David Castle, Ian Caterson, Belinda Chelius, Lyn Chiem, Simon Clarke, Janet Conti, Lexi Crouch, Genevieve Dammery, Natasha Dzajkovski, Jasmine Fardouly, John Feneley, Nasim Foroughi, Mathew Fuller-Tyszkiewicz, Anthea Fursland, Veronica Gonzalez-Arce, Bethanie Gouldthorp, Kelly Griffin, Scott Griffiths, Ashlea Hambleton, Amy Hannigan, Mel Hart, Susan Hart, Phillipa Hay, Ian Hickie, Francis Kay-Lambkin, Ross King, Michael Kohn, Eyza Koreshe, Isabel Krug, Anvi Le, Jake Linardon, Randall Long, Amanda Long, Sloane Madden, Sarah Maguire, Danielle Maloney, Peta Marks, Sian McLean, Thy Meddick, Jane Miskovic-Wheatley, Deborah Mitchison, Richard O’Kearney, Roger Paterson, Susan Paxton, Melissa Pehlivan, Genevieve Pepin, Andrea Phillipou, Judith Piccone, Rebecca Pinkus, Bronwyn Raykos, Paul Rhodes, Elizabeth Rieger, Karen Rockett, Sarah Rodan, Janice Russell, Haley Russell, Fiona Salter, Susan Sawyer, Beth Shelton, Urvashnee Singh, Sophie Smith, Evelyn Smith, Karen Spielman, Sarah Squire, Juliette Thomson, Marika Tiggemann, Stephen Touyz, Ranjani Utpala, Lenny Vartanian, Andrew Wallis, Warren Ward, Sarah Wells, Eleanor Wertheim, Simon Wilksch, Michelle Williams, Phillipa Hay, Jane Miskovic-Wheatley, Stephen Touyz, Sarah Maguire

**Affiliations:** 1 Inside Out Institute, University of Sydney & Sydney Local Health District, Sydney, NSW Australia; 2 Sydney Local Health District, New South Wales Health, Sydney, NSW Australia; 3 Healthcare Management Advisors, Melbourne, VIC Australia; 4 Translational Health Research Institute, Western Sydney University, Sydney, NSW Australia; 5 School of Psychology, Faculty of Science, The University of Sydney, Sydney, NSW Australia; 6 Central Clinical School, Faculty of Medicine and Health, Charles Perkins Centre (D17), University of Sydney, Sydney, NSW 2006 Australia

**Keywords:** Eating disorders, Mental health, Policy, Research, Research translation, Rapid review, Australia

## Abstract

**Background:**

Eating disorders (EDs) are highly complex mental illnesses associated with significant medical complications. There are currently knowledge gaps in research relating to the epidemiology, aetiology, treatment, burden, and outcomes of eating disorders. To clearly identify and begin addressing the major deficits in the scientific, medical, and clinical understanding of these mental illnesses, the Australian Government Department of Health in 2019 funded the InsideOut Institute (IOI) to develop the Australian Eating Disorder Research and Translation Strategy, the primary aim of which was to identify priorities and targets for building research capacity and outputs. A series of rapid reviews (RR) were conducted to map the current state of knowledge, identify evidence gaps, and inform development of the national research strategy. Published peer-reviewed literature on DSM-5 listed EDs, across eight knowledge domains was reviewed: (1) population, prevalence, disease burden, Quality of Life in Western developed countries; (2) risk factors; (3) co-occurring conditions and medical complications; (4) screening and diagnosis; (5) prevention and early intervention; (6) psychotherapies and relapse prevention; (7) models of care; (8) pharmacotherapies, alternative and adjunctive therapies; and (9) outcomes (including mortality). While RRs are systematic in nature, they are distinct from systematic reviews in their aim to gather evidence in a timely manner to support decision-making on urgent or high-priority health concerns at the national level.

**Results:**

Three medical science databases were searched as the primary source of literature for the RRs: Science Direct, PubMed and OVID (Medline). The search was completed on 31st May 2021 (spanning January 2009–May 2021). At writing, a total of 1,320 articles met eligibility criteria and were included in the final review.

**Conclusions:**

For each RR, the evidence has been organised to review the knowledge area and identify gaps for further research and investment. The series of RRs (published separately within the current series) are designed to support the development of research and translation practice in the field of EDs. They highlight areas for investment and investigation, and provide researchers, service planners and providers, and research funders rapid access to quality current evidence, which has been synthesised and organised to assist decision-making.

## Introduction

Eating disorders (ED) are complex mental illnesses associated with numerous physical complications and mental health comorbidities [1–4]. EDs have some of the highest mortality rates of the psychiatric illnesses and carry significant disease burden [5]. There is a lack of consistent research data on this illness group across most knowledge areas including prevalence, prevention and early intervention, outcome (including mortality), models of care, and long-term outcomes [6].

Six primary EDs are defined by the DSM-5 and affect more than 1 million Australians per year [7]. They are Anorexia Nervosa (AN), Bulimia Nervosa (BN), Binge Eating Disorder (BED), Other Specified Feeding or Eating Disorder (OSFED), Avoidant Restrictive Food Intake Disorder (ARFID), and Unspecified Feeding or Eating Disorder (UFED) [8]. Despite having relatively reliable diagnostic categories, there are major deficits in scientific, medical, and clinical understanding of EDs [9].

Specifically, no large-scale review of the field has been conducted that appraises what is currently known about EDs. Consequently, gaps have not been identified in such a way that progresses the field in its focus on areas requiring urgent research attention. Beyond the focus on knowledge gaps, it is also the case in Australia that there has been a lack of development of the research workforce and pipeline for high quality researchers. Lack of student funding (despite high demand for postgraduate research degree supervision [10]) and a general shortage of funded research activity [11] means the sector has historically been led by a handful of dedicated academics usually in teaching or clinical positions with part-time research capabilities.. Nevertheless, Australian publication rates remain paradoxically high relative to the size of the research community [12]. Investment in ED research, even internationally, is markedly lower than that of other major mental illnesses particularly relative to disease burden—for example, in 2020 $1.71 was spent per individual with an ED vs. $5.08 for anxiety, $19.81 for depression, $23.89 for autism, and $197.14 for schizophrenia) [13], with all the intended down-stream impacts on workforce, outputs and breakthroughs.

**Fig. 1:**
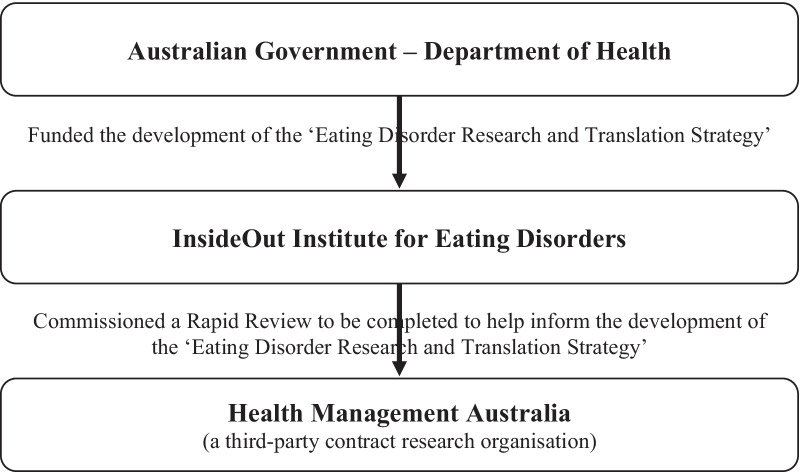
Organisational involvement flow-chart

In acknowledgement of these facts, in 2019 the Australian Government—Department of Health funded the development of the first national Eating Disorder Research and Translation Strategy for Australia, to identify strategic priorities and targets for building research capacity and outputs. The InsideOut Institute (IOI) for EDs (Sydney, Australia) was tasked with developing the strategy. As part of this process, IOI commissioned Health Management Australia (HMA; Victoria, Australia) to conduct an independent scoping of the ED field. The RR process was conducted in consultation with the expert researcher community, to provide a ready and organised summary of the evidence base to help guide research and investment in the field (Fig. 1).

### Aim and research questions

The primary aim of the RRs was to inform the development of a national strategy for EDs. Specifically, the RR was conducted with the objective of reviewing all available peer-reviewed literature on the six DSM-5 listed EDs to support the development of Australia’s national research priorities on EDs.

The specific research questions of the RR were developed using the Population, Intervention, Comparison, Outcome (PICO) framework—an evidence-based framework for generating research questions [14].

**Fig. 2:**
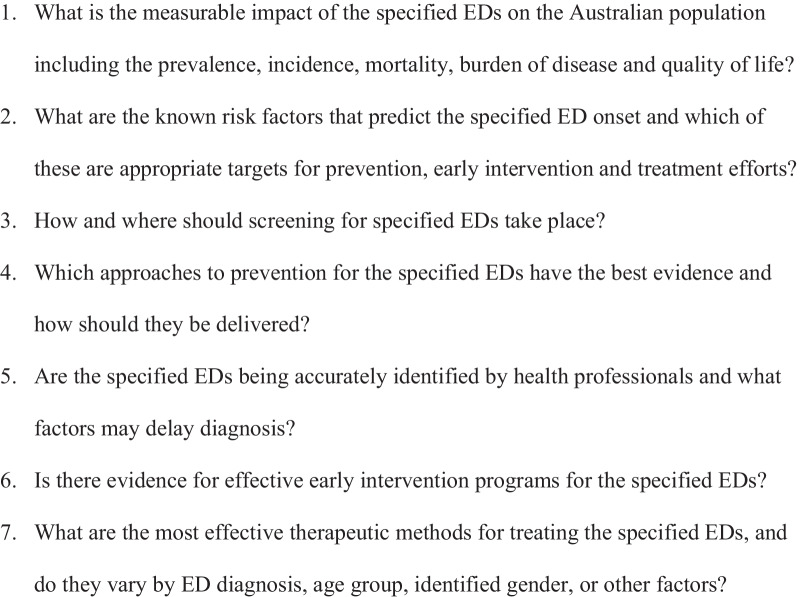
Research questions answered by the rapid review

The research questions are, outlined in Fig. 2.

## Methodology

This InsideOut Institute (IOI) for Eating Disorders commissioned Health Management Australia (HMA), a private third-party specialist health management firm, to conduct a rapid review (RR) to assess the current research base and inform part of the development of the Eating Disorder Research and Translation Strategy (see Fig. 1). It was determined a RR process was best suited for the present purpose. A RR is systematic in nature, and largely adheres to the ‘gold standard’ Preferred Reporting Items for Systematic Reviews and Meta-Analysis (PRISMA) guidelines [15]. Compared to a traditional systematic review, a RR is often conducted with broader search terms and inclusion criteria, attempting to understand a field of study in its entirety and producing a larger number of returned search results [16–18]. According to the Cochrane Rapid Reviews Interim Guidelines [19], RRs are predominately used as a time-sensitive means of producing evidence for decision-making processes in order to address high-priority or urgent health concerns. This is achieved by streamlining specific methods (such as searching fewer, more general, databases) and involving key stakeholders—through ongoing consultations and feedback—in determining the research question/s to be answered and defining the eligibility criteria and outcomes to be examined [19]. Thus, RRs may provide a snapshot of key findings that detail the current state of a field and can, in turn, strategically inform decision-making around policy, offering directions for future research [19]. This is particularly useful within the context of setting national research priorities in a collaborative, unified, and unbiased manner. Moreover, the approach outlined in the current paper offers a unique opportunity utilising worldwide eating disorder experts and researchers, from Australia, which mitigates, to some extent, the possibility of missing key information in the field.

### Eligibility criteria for inclusion of studies

To establish a broad understanding of the progress made in the field of EDs, and to capture the largest evidence base from the past 12 years (originally 2009–2019, but expanded to include the preceding two years), the eligibility criteria for included studies into the rapid review were kept broad. Specifically,

Included articles were published:

- Between January 2009 and May 2021;
- Peer-reviewed; and,
- in English.

Further, the search methodology was designed to capture all relevant peer-reviewed literature, with a focus on high-level evidence studies such as: meta-analyses; systematic reviews; moderately sized randomised controlled studies (RCTs) (n > 50); small to moderately sized controlled-cohort studies (n > 50), or population studies (n > 500).

**Fig. 3:**
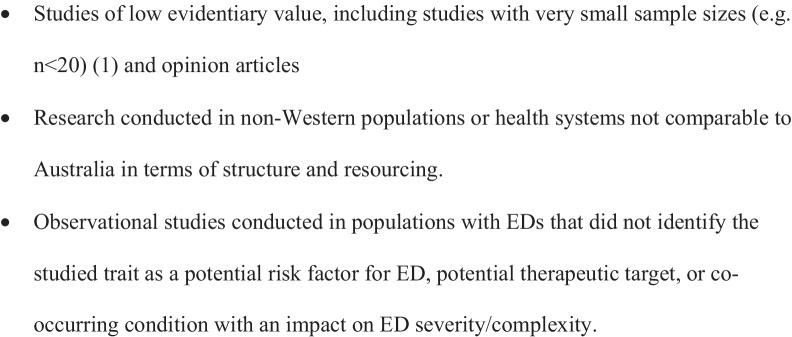
Exclusion criteria

Exclusion criteria for articles are outlined Fig. 3.

The diagnoses ARFID and UFED necessitated a less stringent eligibility criterion due to a paucity of published articles. In focus areas where the evidence base is emerging and fewer studies have been conducted, smaller studies (n =  < 20) and narrative reviews were also considered and included.

### Information sources

Three medical science databases were searched as the primary source of literature for the RR: Science Direct, PubMed and OVID (Medline). This approach facilitated a comprehensive coverage of available publications as well as the filtering of results by additional search parameters to keep track of the overall number of results.

Additional articles were identified using citation links and hyperlinks for related articles in databases. Manual searching of reference lists was conducted for emerging research areas (such as ARFID and UFED). Any eligible studies related to a specific topic covered by the RR may also be identified by the expert researchers involved in drafting forthcoming manuscripts. Additional studies will be clearly identified in the respective paper and reported on accordingly.

### Search strategy

Search terms entered into the databases yielded a result if the term was included in one or more of the following article identifiers: title; abstract; author-specified key words; journal title, or author name/affiliations. The search was conducted by three reviewers from HMA (led by AL) between 5 December 2019 and 16 January 2020.

Based on feedback from researchers, experts, lived-experience advocates, and carers (constituting the *National Eating Disorder Research Consortium, henceforth the ‘consortium’*), an additional search was conducted between 6 May 2020 and 11 May 2020 using the key search term ‘eating disorder’ in conjunction with the keywords outlined in Fig. 3. Furthermore, an updated search was run for all key search terms between 11 May 2020 and 30th May 2021. Any additional studies identified by expert researchers as being relevant were screened for eligibility and discussed in the respective paper. The objective of additional searches is to ensure all up-to-date literature meeting eligibility is included in the review prior to publication of results.

Initial screening of articles based on their titles/abstracts was conducted by three independent HMA reviewers (led by AL) as part of the search strategy process. Articles assessed for inclusion based on the criteria underwent a further review process based on the evidence presented with relevance for the RR – this was conducted by two of the HMA reviewers (AL + colleague) involved in initial screening. Discrepancies were resolved by discussion between the reviewers with disputes referred to an expert research panel (ST, PH, SP) for final decision. Evidence presented in the RR is based on literature that satisfied criteria following this subsequent review process.

#### Key search terms

A broad search strategy was applied to facilitate a comprehensive search of the literature by each specified in-scope ED diagnosis. Key search terms were identified through preliminary searches and in consultation with members of the consortium and expert research panel.

The review had a translational research focus with the objective of identifying evidence relevant to developing optimal care pathways. Searches therefore used a Population, Intervention, Comparison, Outcome (PICO) approach to identify literature relating to population impact, prevention and early intervention, treatment, and long-term outcomes [20]. The PICO framework is widely used to develop research questions that are clinically translatable, allowing for a focused and comprehensive search of a breadth of relevant literature [20]. It involves clearly outlining the population or illness group of interest, outlining the intervention or therapies of interest, stating clearly if there are other groups being compared to the population chosen, and clearly defining which outcomes are to be examined.

**Fig. 4:**
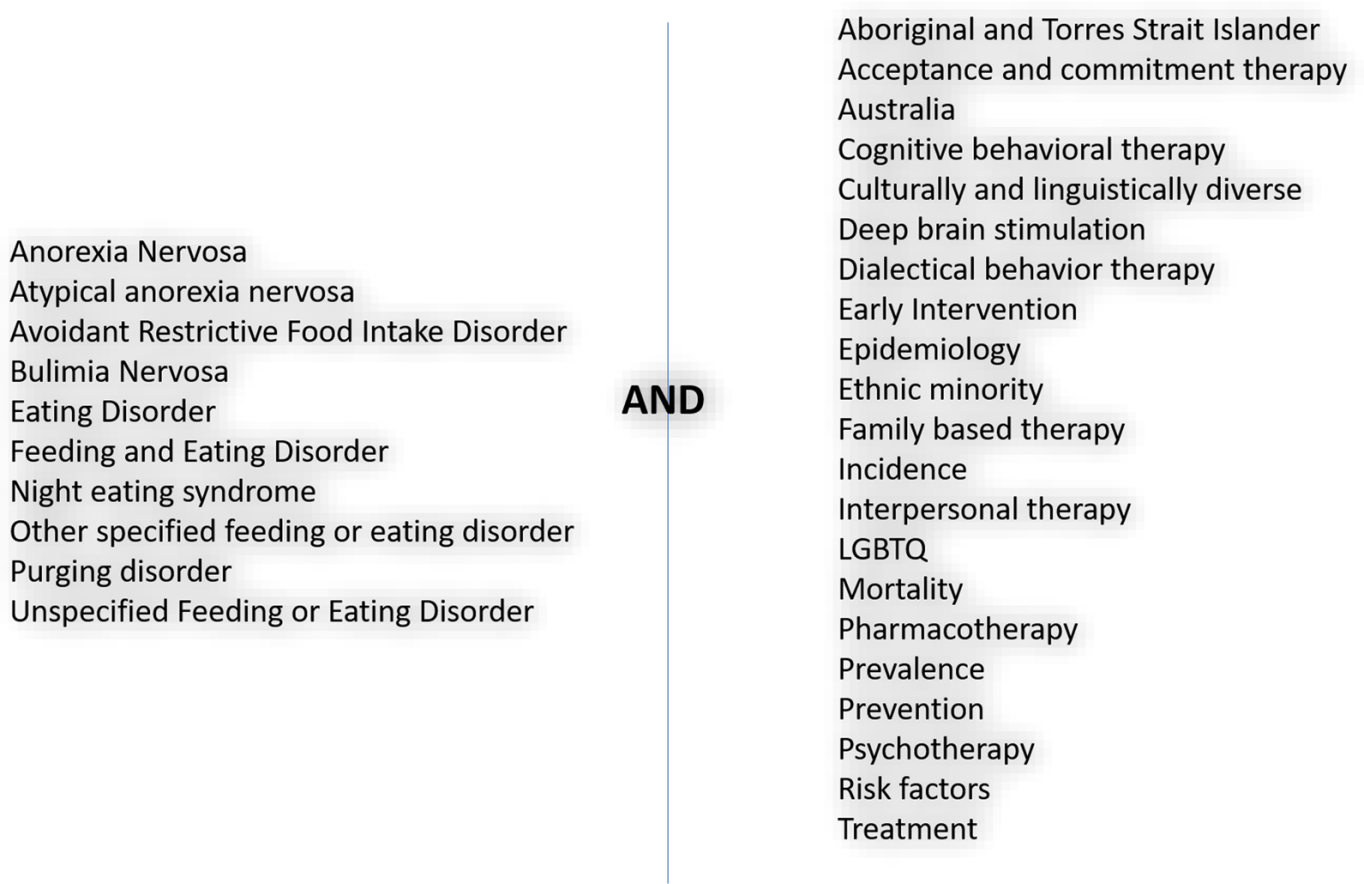
Keyword search terms. *Note*: Where applicable all search terms utilised wildcard (*) and combination searches (database permitting) to ensure all variations of a term were captured. Terms in either the ‘disorders’ column or ‘topic specifier’ column were separated with the ‘OR’ Boolean operator

A total of 30 keywords were used across each of the chosen databases. A list of the keywords is shown in Fig. 4.

### Study records

#### Data management and data collection process

Data were managed by HMA in accordance with their data management policies. Additional information may be found on the HMA website (hma.com.au). The data collection process by HMA included entering studies into an independent database, including key study information.

#### Selection process

Studies were selected based upon the agreed inclusion/exclusion criteria developed by the RR project team and authors (SM, PM, PH, ST) and HMA. Selection of evidence for inclusion was conducted by two reviewers from HMA. Reference lists were manually screened for emerging areas of research that had limited literature—this process also involved the Expert Research Collaborative suggesting additional references that may have not been identified previously.

Due to the lack of research on newer DSM-5 [8] disorders, which were not present in DSM-IV [21], a large proportion of included studies relating primarily to UFED and ARFID were narrative reviews, used uncontrolled designs, or included smaller sample sizes (n =  < 20) than those conducted in individuals with AN, BN and BED.

### Risk of bias

#### Individual studies

As the overarching purpose of this review was to inform an Australian Research and Translation Strategy, selection of studies focused on information and interventions that could be readily applied to the Australian population. Therefore, some exploratory studies or studies investigating emerging illnesses -such as ARFID and UFED, with sample sizes smaller than 20 were included. As noted in the discussion, this may introduce a risk of bias [22].

Where possible, results on the efficacy of interventions included information reported in studies on the length of patient follow-up. Studies where results were compared to a control or active comparator were also identified.

#### Data synthesis

The cumulative evidence of each of the studies, categorised under relevant subject headings, was synthesised at a high level indicating whether there was consensus in the field regarding the validity of hypotheses, benefit of treatments or interventions, and the impact of EDs on the population.

Given the heterogeneity of studies and the scope of the RR, no meta-analysis was conducted; instead, an evidence synthesis approach was utilised.

## Results

In total, 1320 articles were included in the RR. Figure 4 presents the PRISMA four-phase flow diagram detailing the data collection process and number of returned search results in each phase.

Full article reviews were conducted on 1968 publications with a final subset of 1308 studies included in the RR. Upon review of the initial subset by the Expert Research Collaborative, an additional 12 articles were identified for inclusion, bringing the number of included articles at the time of writing this methods paper to 1320.

**Fig. 5:**
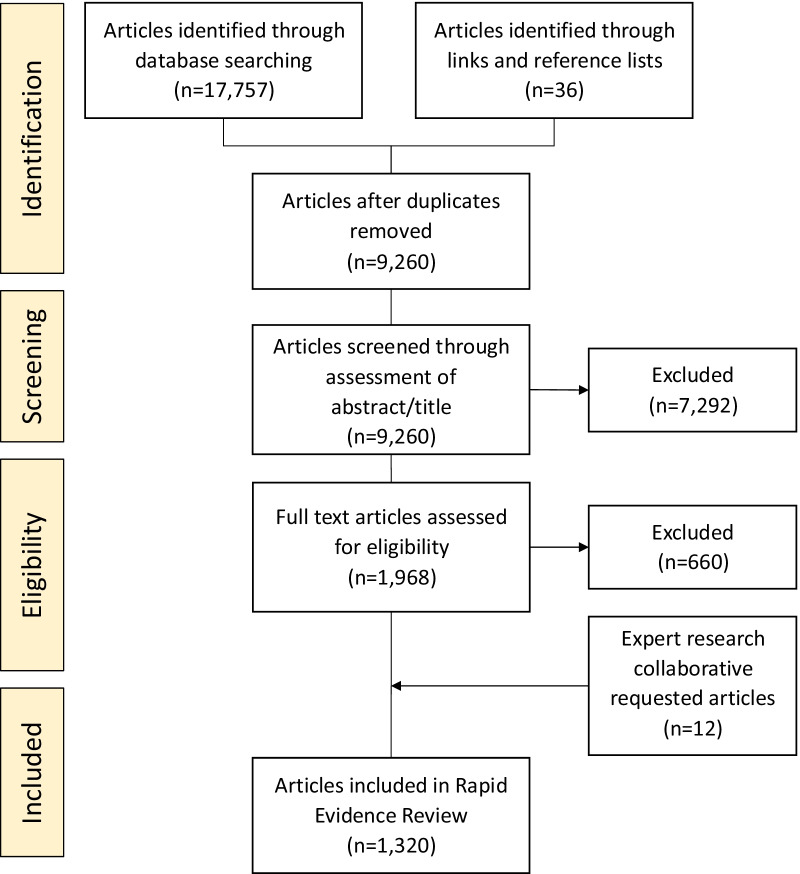
PRISMA flow-chart of search results

To inform the development of new knowledge in EDs, identified information was grouped to represent broad focus areas—epidemiology, risk factors, screening and diagnosis, early intervention, comorbidities, treatment and models of care, and disorder management and outcomes. Results from the search fell into these seven broad areas displayed in Fig. 5. As mentioned, the RR included a translational research focus, potentially contributing to a large proportion of the studies being related to treatments and models of care. The largest proportion of evidence was related to ED risk factors (20%).

Only a small proportion of studies relating to screening and diagnosis (6%) and models of care (4%) were identified. As this represented only 10% of the evidence base identified in the review, this suggests there is a gap in knowledge in these areas.

### Individual study results

**Fig. 6:**
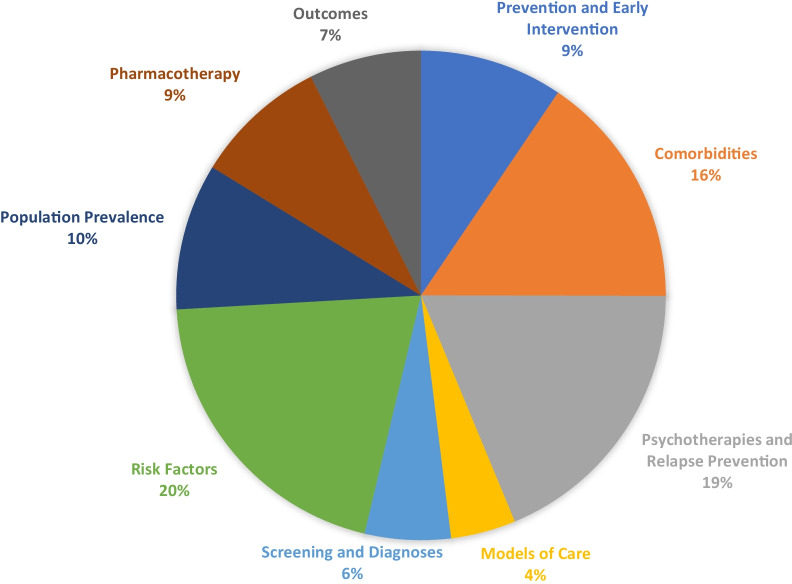
Broad categorisation of evidence base identified

Given the number of search results returned, individual findings for each of the broad categories presented in Fig. 6 will be reported in individual forthcoming publications. These papers will span the following areas: 1) population, prevalence, disease burden, Quality of Life (QoL) in Western developed countries; 2) risk factors; 3) co-occurring conditions and medical complications; 4) screening and diagnosis; 5) prevention and early intervention; 6) psychotherapies and relapse prevention; 7) models of care; 8) pharmacotherapies, alternative and adjunctive therapies; and 9) outcomes (including mortality).

### Distribution of the included literature (descriptive)

Of the included papers, over two thirds (77%) were published following publication of the DSM-5, however the proportion of studies adopting the updated criteria was not examined. Of the nine identified categories, 131 (10%) included studies examining population, prevalence, disease burden, and QoL (epidemiology); 278 (20%) investigated risk factors (aetiology); 214 (16%) looked at co-occurring conditions and medical complications (comorbidities); 77 (6%) focused on screening and diagnosis (diagnostics); 129 (9%) explored aspects of prevention and early intervention (intervention); 257 (19%) probed elements of psychotherapies and relapse prevention (treatment); 59 (4%) considered models of care (care models); 121 (9%) researched pharmacotherapies, alternative and adjunctive therapies (treatment); and 102 (7%) measured outcomes. However, it should be noted that around 3% (n = 46) of studies firmly fell into more than one of the nine categories.

Several trends around the general distribution of the literature, as they relate to the above nine categories, were noted. Many of the included epidemiological studies were based on US and European samples and focused on the ‘core’ eating disorders of Anorexia Nervosa, Bulimia Nervosa, and Binge Eating Disorder. Similarly, there appeared a large evidence-base examining risk factors associated with these core eating disorders. However, little evidence was identified examining the risk factors of Other Specified Feeding and Eating Disorders (OSFED) and Avoidant/ Restrictive Food Intake Disorder (ARFID). Genetics, the environment, and other psychosocial influences were found to all contribute in part to eating disorder development.

Included studies examining co-occurring conditions primarily considered psychological comorbidities, with an abundance of studies investigating anxiety and Obsessive Compulsive Disorder in individuals with eating disorders. Anorexia Nervosa appeared to be among the most widely studied eating disorder group for comorbidities.

Much of the literature identified focused on screening in specific settings, such as universities, colleges, and schools. There was a stark lack of peer-reviewed evidence focused on screening eating disorders in males (n = 1). Further, it was found that while studies on screening and diagnosis are available, there are deficits in available literature that focus on screening and diagnosis in primary care settings, among women accessing fertility and reproductive health services, obese individuals, and adolescents with diabetes. Evidence suggests a need for increased screening for EDs in primary care settings.

Comparatively few early intervention studies were identified by the review, with some larger studies arising from the USA and UK. There appeared to be lack of consensus between studies on one leading approach to developing and delivering early intervention, however it was noted, broadly, that internet-based programs may have some promise for particular types of EDs. It was also noted that there was a dearth of research on targeted prevention strategies for minority and high-risk groups.

Several implementation studies were identified delivering treatments through health system models of care, conducted in ‘real world’ settings. However, this did not represent a significant proportion of evidence identified. Gaps in services, delayed diagnosis, and subsequently specialist care and treatment were identified to not only lead to poorer health outcomes for individual with eating disorders but also significant government healthcare expenditure. Most treatments explored in by the review included studies focused on behavioural therapies, however findings did not lead to the identification of one leading effective psychotherapy for the treatment of eating disorders, particularly in adults with restrictive type eating disorders. Further, there appears to be little-to-no consensus on the focus for developing approaches to reduce exceedingly high relapse rates.

The evidence-base for treatment of eating disorders with pharmacotherapy is sparse in comparison to psychotherapies. Many studies which examined the use of drug therapies were smaller clinical trials, often without a control group. Included studies therefore contained few Randomised Control Trials (RCTs) that assessed pharmacological interventions specifically for the treatment of eating disorders. Overall, the results indicate some conflicting evidence for atypical antipsychotics for weight-restoration in individuals with restrictive-type eating disorders; however, it should be noted that this is an emerging area of research.

Finally, it was noted that many studies exploring outcomes focused on immediate rather than long-term outcomes post-treatment. Mortality because of an eating disorder, particularly in adults with chronic illness, was found to be a significant outcome which, in general, required further research.

The forthcoming series of papers will explore the above topics and in greater detail.

## Discussion

The current methodological paper outlines the process followed to conduct a set of Rapid Reviews (RR) in the field of EDs. The information identified by the RRs informed development of the first Australian Eating Disorders Research and Translation Strategy 2021–2031. In total, 1,322 studies were eligible for inclusion in the RRs, with included studies being grouped into broad categories. Risk factors (20%), psychotherapies (19%) and comorbidities (16%) made up a majority of the extant literature; followed by population prevalence population, prevalence, disease burden and quality of life (epidemiological) studies (10%); pharmacotherapies (9%) and prevention and early intervention (9%), which was comparatively low given evidence to suggest early intervention—particularly in young people—can significantly improve outcome trajectory [23]. There was limited research on outcomes (7%), screening and diagnosis (6%), and models of care (4%).

The distribution of themes identified by the review highlighted a disproportionate focus on several core eating disorders, namely Anorexia Nervosa, Bulimia Nervosa, and Binge Eating Disorder, with evidence on atypical (e.g., OSFED) or less common presentations (e.g., ARFID)—emerging more slowly. While this may be expected given the relatively ‘recent’ (2013) shift in eating disorder classification in the DSM-5 [8], it highlights that a significant proportion of eating disorder presentations are poorly understood. This may lead to compromised or suboptimal clinical care or treatment outcomes as existing diagnostic tools and treatments are retrofitted to lesser understood presentations—for example similar, or slightly modified, treatments for anorexia nervosa being applied to ARFID [24–26] rather than developed specifically for that diagnostic presentation.

Nonetheless, as the current series focuses on the evidence base as it relates to developing Australia’s National Eating Disorders Research and Translation Strategy, there emerges an interesting picture specific to the Australian context. First and foremost, is the need to assess and determine a 'truer' and more accurate epidemiological understanding of eating disorders in Australia. The estimates put forth in some of the included studies may be considered conservative given the findings of this review point to an underrepresentation of some minority and at-risk groups [27, 28].

Of the included studies, > 15% involved Australian researchers. Australia is producing high-quality and important research in line with other Western, Educated, Industrialised, Rich, and Democratic (WEIRD) countries, however the review suggests there is great need for novel research specifically addressing significant knowledge gaps in the field; particularly, relating to models of care, screening and diagnosis, outcomes (notably long-term outcomes and mortality), pharmacological approaches and prevention and early intervention. These gaps will be explored in further detail throughout the forthcoming series.

### Strengths and limitations

Utilising a RR methodology allowed for the expedient summation and synthesis of the evidence base as it exists currently in the field of EDs to guide the development of a national strategy. The consortium consisted of experts with international reputations in EDs, who contributed to the RR methodology, including search strategy development, alongside IOI and HMA to ensure that the RRs were conducted with integrity and was underpinned by specialist knowledge. Further, the RR methodology allows for well-informed and co-ordinated decision-making and policy implementation support, with evidence able to be provided in an easily accessible manner to policy makers and government agencies. Still, several limitations are apparent. Exploratory studies, or those investigating novel therapies with sample sizes of n < 20, were excluded, excepting for ARFID and UFED due to the minimal evidence available for these diagnoses. Therefore, it is possible some findings emerging from studies with very small samples are not captured by the RRs and that the evidence base for emerging areas may not be as robust in its quality. While the three major medical databases were searched, smaller discipline-specific databases not included in the search may have yielded some additional eligible studies. A period of 12 years was examined (originally 2009–2019, then extended until 2021) meaning that studies outside this time period would not have been deemed eligible. However, all RR topic areas had systematic reviews and meta-analyses included that would have addressed much of the prior literature.

Limiting the included studies to English only, not considering animal model studies that may lead to the development of testable hypotheses, and not specifically including qualitative studies nor grey literature may have also limited the breadth and richness of the data reported in the RRs.

### Significance

The Australian Eating Disorders Research and Translation Strategy (2021–2031) was a multi-layered, multi-phased initiative led by IOI, co-designed with key stakeholders, including people with a lived experience (consumers, carers), researchers, and clinicians. The overall purpose of the strategy is to build a strong research culture that generates innovative co-designed research that transforms practice, informs policy, and meaningfully impacts the wellbeing of all people at risk of developing or living with an ED, their families, and supports. The significance of the RR lies in its potential to identify gaps and inform research priorities, to encourage investment in this under-researched area, and ultimately transform care pathways for this potentially deadly and devastating illness group. By understanding the different areas of ED research, a clear picture to guide government and stakeholders for informed decisions may be achieved. Furthermore, these reviews provide up-to-date knowledge on EDs easily accessible to individuals, organisations and institutions, and governments looking to understand the state of the field.

## Conclusions

Based on a broad search strategy, the findings of the current RRs may be used to further inform and develop policy, practice, and sound evidence-based research, solidifying Australia’s position as a global leader in ED care. The resulting RR papers focusing on: population, prevalence, disease burden, QoL in Western developed countries; risk factors; co-occurring conditions and medical complications; screening and diagnosis; prevention and early intervention; therapy and relapse prevention; models of care; and outcomes, will cover a broad area of the ED field and offer a meaningful synthesis of knowledge to help build upon into the future. By utilising a RR methodology, a current and thorough overview of the ED field is established to guide and develop policy, care, and research.

## Data Availability

No data is associated with this manuscript

## Abbreviations

A-AN: Atypical Anorexia Nervosa
AN: Anorexia Nervosa
AN-BP: Anorexia Nervosa binge/purge subtype
ARFID: Avoidant Restrictive Food Intake Disorder
BED: Binge Eating Disorder
BMI: Body Mass Index
BN: Bulimia Nervosa
CALD: Culturally and Linguistically Diverse
CBT: Cognitive Behavioural Therapy
DBT: Dialectic Behaviour Therapy
ED: Eating Disorder
FBT: Family-based therapy
HMA: Healthcare Management Advisors
HRQoL: Health Related Quality of Life
IOI: InsideOut Institute
IPT: Interpersonal therapy
OSFED: Other Specified Feeding or Eating Disorder
QoL: Quality of Life
RR: National Eating Disorder Research & Translation Strategy Rapid Review
UFED: Unspecified Feeding or Eating Disorder

## Declarations

### Adherence to PRISMA guidelines

The current manuscript adheres to PRISMA guidelines for the development and reporting of review protocols [29]. *Update*: The current protocol is not related to a previously published review; *Registration*: The current protocol has not been submitted to a review registry (e.g., PROSPERO); *Amendments*: Any changes to the methodology outlined in the current protocol will be documented explicitly in the resulting relevant publications.
